# Supplementary material for: The interactome of intact mitochondria by cross-linking mass spectrometry provides evidence for coexisting respiratory supercomplexes
Source: Mol Cell Proteomics. 2017 Dec 8;17(2):216–32. doi: 10.1074/mcp.RA117.000470 (PMC5795388; doi:10.1074/mcp.RA117.000470)
Supplement: Supplemental Data [file supp_RA117.000470_133922_0_supp_23978_fzffwf.zip › spectra_annotation/mito_spectra_annotation/159-1-12-1-6-1.pdf]

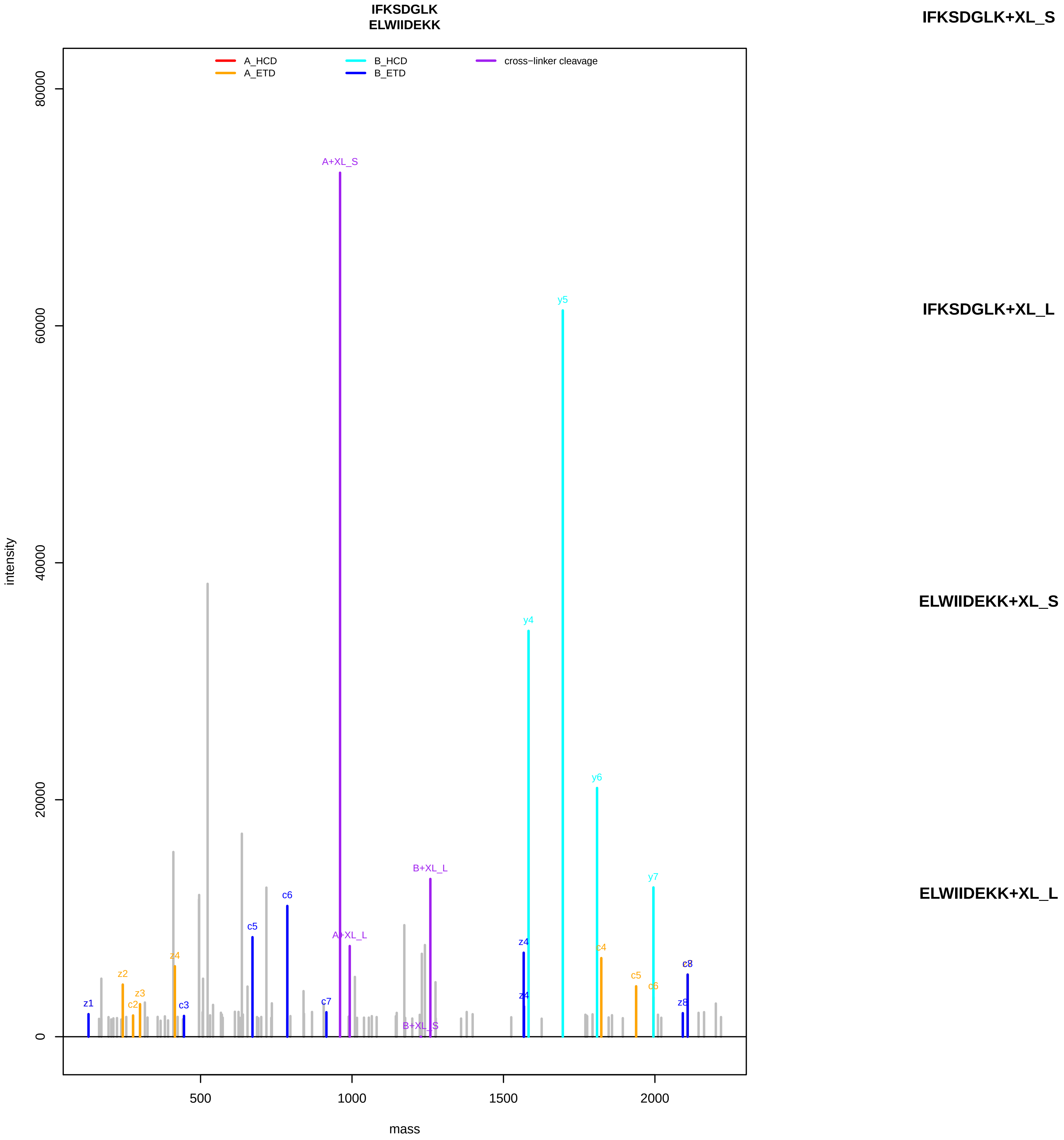
IFKSDGLK
ELWIIDEKK
A_HCD
A_ETD
B_HCD
B_ETD
cross−linker cleavage
A+XL_S
y5
y4
y6
B+XL_L
y7
c6
c5
A+XL_L
z4
c4
c5
c6
c8
z4
z2
c2
z3
z1
c3
c7
z8
B+XL_S
intensity
mass
0
20000
40000
60000
80000
500
1000
1500
2000
IFKSDGLK+XL_S
IFKSDGLK+XL_L
ELWIIDEKK+XL_S
ELWIIDEKK+XL_L
